# Learning and Memory Alterations Are Associated with Hippocampal *N*-acetylaspartate in a Rat Model of Depression as Measured by ^1^H-MRS

**DOI:** 10.1371/journal.pone.0028686

**Published:** 2011-12-14

**Authors:** Guangjun Xi, Jiaojie Hui, Zhijun Zhang, Shanshan Liu, Xiangrong Zhang, Gaojun Teng, Kevin C. Chan, Ed X. Wu, Binbin Nie, Baoci Shan, Lingjiang Li, Gavin P. Reynolds

**Affiliations:** 1 Department of Neurology, Affiliated ZhongDa Hospital and the Institution Neuropsychiatry of Southeast University, Nanjing, Jiangsu, China; 2 Institution of Molecular Imaging, Southeast University, Nanjing, Jiangsu, China; 3 Laboratory of Biomedical Imaging and Signal Processing, The University of Hong Kong, Pokfulam, Hong Kong, China; 4 Department of Electrical and Electronic Engineering, The University of Hong Kong, Pokfulam, Hong Kong, China; 5 Key Laboratory of Nuclear Analytical Techniques, Institute of High Energy Physics, Chinese Academy of Sciences, Beijing, China; 6 Mental Health Institute, The Second Xiangya Hospital of Central South University, Changsha, Hunan, China; 7 Biomedical Research Centre, Sheffield Hallam University, Sheffield, United Kingdom; Chiba University Center for Forensic Mental Health, Japan

## Abstract

It is generally accepted that cognitive processes, such as learning and memory, are affected in depression. The present study used a rat model of depression, chronic unpredictable mild stress (CUMS), to determine whether hippocampal volume and neurochemical changes were involved in learning and memory alterations. A further aim was to determine whether these effects could be ameliorated by escitalopram treatment, as assessed with the non-invasive techniques of structural magnetic resonance imaging (MRI) and magnetic resonance spectroscopy (MRS). Our results demonstrated that CUMS had a dramatic influence on spatial cognitive performance in the Morris water maze task, and CUMS reduced the concentration of neuronal marker *N*-acetylaspartate (NAA) in the hippocampus. These effects could be significantly reversed by repeated administration of escitalopram. However, neither chronic stress nor escitalopram treatment influenced hippocampal volume. Of note, the learning and memory alterations of the rats were associated with right hippocampal NAA concentration. Our results indicate that in depression, NAA may be a more sensitive measure of cognitive function than hippocampal volume.

## Introduction

Depression, with 10 to 20% lifetime prevalence, is the most common psychiatric illness that involves the disturbance of mood [1]. It is becoming increasingly clear that disturbances in cognitive processes, especially the impairment of learning and memory ability, play an important role in the development and maintenance of depression [2]–[6]. However, the mechanisms are not clearly understood.

The hippocampus is a brain structure that has been extensively studied with regard to stress, depression and the effects of antidepressants. Previous studies have revealed that the hippocampal formation is very susceptible to stress-induced functional and morphological alterations, including a reduction in the expression of brain-derived neurotrophic factor (BDNF) [7], decreases in long-term potentiation [8]–[9] and inhibition of neurogenesis in the dentate gyrus (DG) [10]. Accordingly, functional neuroimaging studies of depressed individuals have identified abnormalities of resting blood flow and glucose metabolism in the hippocampus and brain regions that are extensively connected to it [11]. Additionally, for many years, the hippocampus has been thought to play a very important role in encoding, consolidating and processing new information leading to learning and memory in both animals and humans [12]–[15]. To further our understanding of this evidence, it is very important to investigate whether stress-induced functional and morphological changes in the hippocampus are correlated with impairments in learning and memory in depression.

To date, clinical studies have failed to account for depressive burden, treatment history, age at onset of depression and heterogeous causes of depression. For this reason, animal studies are indispensable to improving our understanding of the neurobiological processes that underlie depression, depression-cognition interactions, and antidepressant effects. In the present study, a regimen of chronic unpredictable mild stress (CUMS), which was initially described by Willner [16] and termed chronic mild stress (CMS), was used as a reliable experimental model of depression [17]–[18]. Most importantly, CUMS has been shown to mimic daily hassles and stress levels in humans. Thus, this regimen provides a relatively realistic animal model of anhedonia, a core symptom and hallmark of depressive disorder as defined in the diagnostic and statistical manual of mental disorders IV (DSM-IV) [19].

Until recently, research has focused on the learning and memory impairments associated with depression [5], [20]–[21], with little examination of the relationship between the neurochemical consequences of CUMS or antidepressant treatment and the behavioral changes. Magnetic resonance spectroscopy (MRS), which has become a versatile tool in medicine and pharmacological research, is a unique method for non-invasive quantification of brain metabolites such as *N*-acetylaspartate (NAA) which plays a role in myelinisation and neuronal energy metabolism and is interpreted as a marker of neuron density and function, creatine and phosphocreatine (Cr) as a measure of cellular energy stores and usually taken as the internal standard for comparison for its relative constance, choline-containing compounds (Cho) as a measure of membrane turnover, myo-inositol (MI) as an astrocyte marker and glutamate (Glu) as a measure of glutamate metabolism [22]. Recent technical and methodological developments in high field and in spectral analysis have dramatically increased the neurochemical information content achievable from in vivo MRS spectra [23]–[24].

In the present study, we aimed to use the non-invasive techniques of structural magnetic resonance imaging (MRI) and MRS to investigate whether the hippocampal volume and neurochemical changes would be related to the learning and memory changes induced by CUMS or escitalopram treatment in a rat model of CUMS.

## Results

### CUMS-induced effects on depressive-like behaviours and learning and memory are reversed by chronic escitalopram treatment

**Figure 1:**
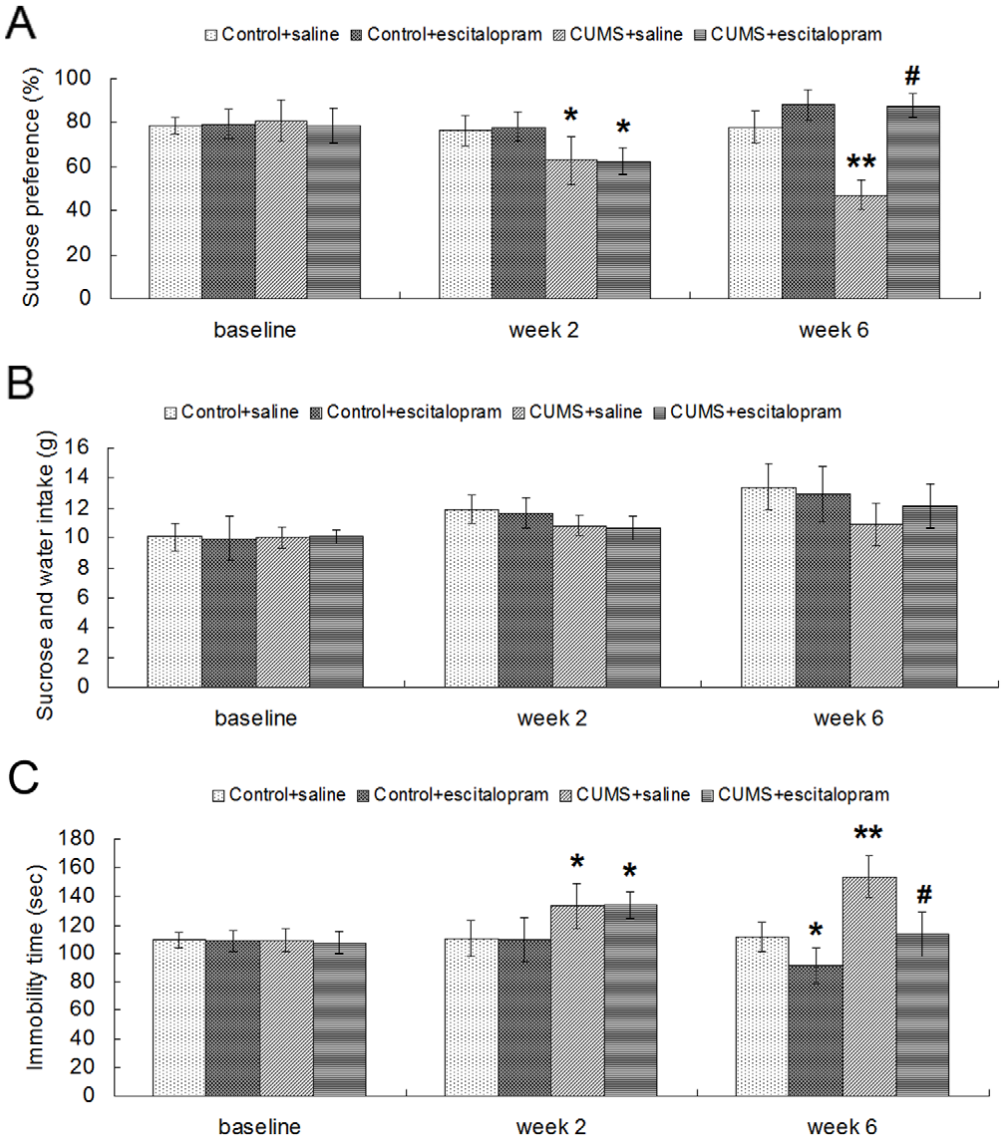
Effects of CUMS and escitalopram treatment on sucrose preference (A), sucrose and water total intake (B) and immobility time in forced swimming test (C) Data are presented as mean ± S.D. (n = 6 per group). * *P*<0.05, ** *P*<0.01 vs. control+saline group, # *P*<0.01 vs. CUMS+saline group.

At the beginning of the experimental procedure, there were no significant differences among the groups exposed to the sucrose preference test [F(3, 20) = 0.138, *P* = 0.936] and the forced swimming test [F(3, 20) = 0.159, *P* = 0.694]. After CUMS for 2 weeks, stressed rats (CUMS+saline and CUMS+escitalopram groups) showed a significant decrease in sucrose preference [F(3, 20) = 6.997, *P* = 0.002] and a significant increase in immobility time [F(3, 20) = 13.666, *P* = 0.001]. At the end of the 6th week, two-way analysis of variance (ANOVA) (CUMS×escitalopram) revealed significant effects of CUMS [F(1, 22) = 32.602, *P* = 0.000], escitalopram [F(1, 22) = 85.595, *P* = 0.000] and a CUMS×escitalopram interaction [F(2, 21) = 31.222, *P* = 0.000] on the sucrose preference. Post-hoc analysis showed that the sucrose preference of CUMS+saline animals was significantly decreased compared with control+saline rats (*P*<0.05), and this was reversed by chronic escitalopram administration (*P*<0.05) (Fig. 1A). There was no significant difference among groups on total intake of sucrose and water at the 3 time points (Fig. 1B). In the forced swimming test, similar effects of CUMS [F(1, 22) = 34.315, *P* = 0.000] and escitalopram [F(1, 22) = 30.515, *P* = 0.000] were demonstrated on the immobility time, with longer immobility in CUMS+saline (*P*<0.05, compared with control+saline group) and a reversal of this effect in CUMS+escitalopram (*P*<0.05, compared with CUMS+saline group) (Fig. 1C).

**Figure 2:**
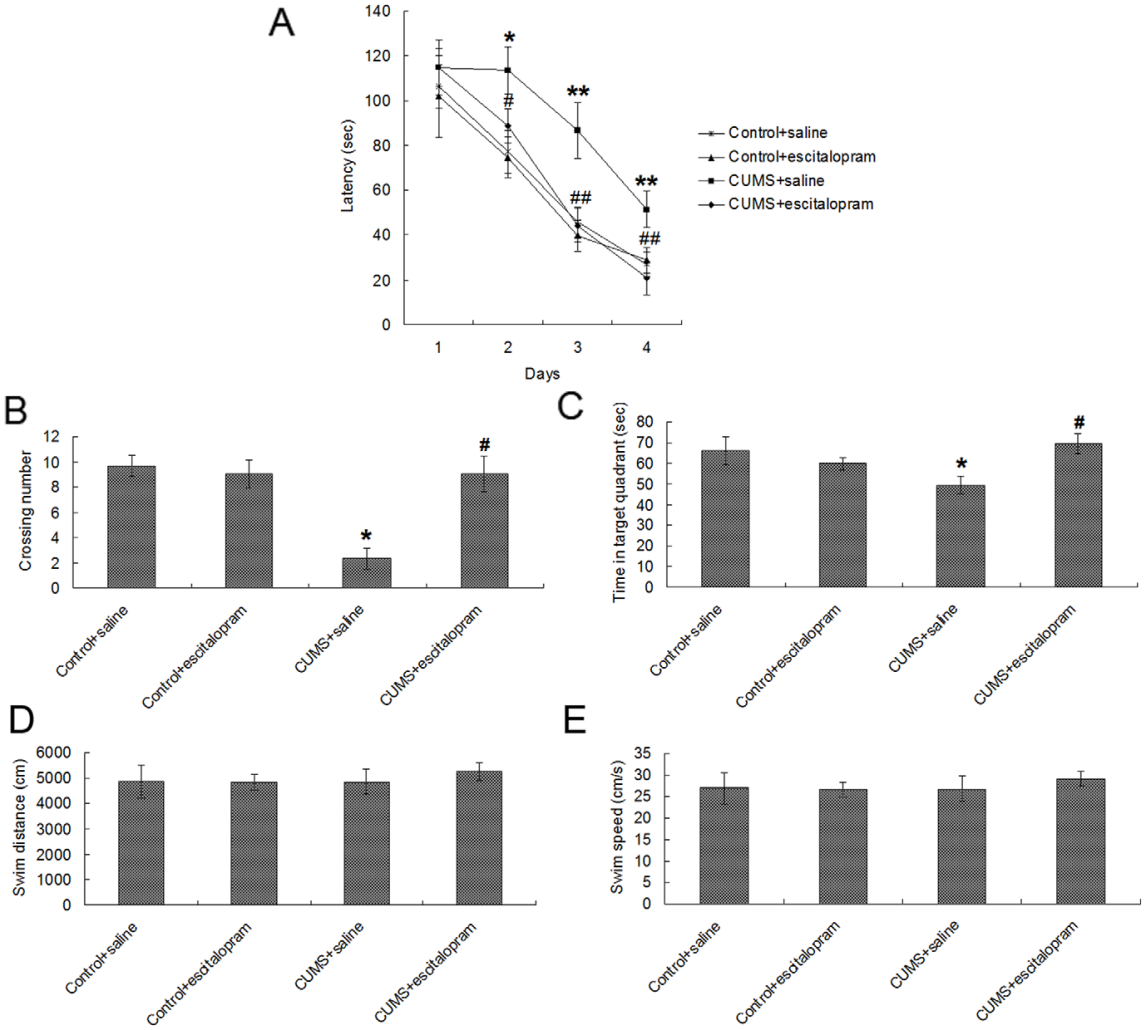
Effects of CUMS and escitalopram treatment on acquisition and consolidation in the Morris water maze test. (A) In the acquisition trials, CUMS rats showed longer escape latency during training days 2–4, while escitalopram treatment restored the CUMS-induced longer latencies to control levels. In the probe trial, CUMS impaired memory retrieval as indicated by fewer crossing times of the platform position (B) and less time spent in the target quadrant (C), while escitalopram treatment restored the CUMS-induced impairment of memory retrieval to levels seen in controls. There was no significant difference of swim distance (D) and swim speed (E) among groups. Data are presented as mean ± S.D. (n = 6 per group). * *P*<0.05, ** *P*<0.01 vs. control+saline group. # *P*<0.05, # # *P*<0.01, vs. CUMS+saline group.

Fig. 2A shows the average escape latency onto a hidden platform in the acquisition trials of the Morris water maze test, with progressively shorter latency on consecutive days. Overall, there was a significant effect of day [F(3, 20) = 259.487, *P* = 0.000], CUMS [F(1, 22) = 169.387, *P* = 0.000] and escitalopram [F(1, 22) = 112.609, *P* = 0.000] on latency to find the platform. A further day-by-day analysis revealed that the CUMS+saline group latencies were significantly longer than the other three groups on day 2 (*P*<0.05), day 3 (*P*<0.01) and day 4 (*P*<0.01), whereas no significant difference in swimming velocity was observed among the different experimental groups.

In the probe trial of the Morris water maze test, CUMS had a significant effect on crossing times [F(1, 22) = 71.176, *P* = 0.000], but no significant effect on the time in target quadrant [F(1, 22) = 3.241, *P* = 0.087], while escitalopram had a significant effect on both crossing times [F(1, 22) = 47.647, *P* = 0.000] and the time in target quadrant [F(1, 22) = 11.688, *P* = 0.003]. Compared with the control+saline group, CUMS+saline rats displayed fewer crossings (*P*<0.05) and less time swimming in the target quadrant (*P*<0.05). Escitalopram restored these changes to control levels (Fig. 2B and 2C). During the period of memory retrieval, the swim distance and swim speed were similar among groups (Fig. 2D and 2E).

### CUMS-induced effects on hippocampal volume and neurochemistry are reversed by chronic escitalopram treatment

To account for variations in brain size, hippocampal volume was normalized to intracranial volume (ICV). The normalized hippocampal volume of each group was provided as Table 1. Stress and escitalopram did not influence the volume of hippocampus either in the left [stress, F(1, 22) = 0.027, *P* = 0.871; escitalopram, F(1, 22) = 0.096, *P* = 0.760] or the right [stress, F(1, 22) = 0.185, *P* = 0.673; escitalopram, F(1, 22) = 0.166, *P* = 0.689] side.

**Table 1:** Effects of CUMS and escitalopram treatment on normalized hippocampal volume.

Laterality	CON+saline	CON+ESC	CUMS+saline	CUMS+ESC
Left	30.2±3.4	29.6±2.8	29.4±2.3	30.9±3.4
Right	28.8±4.1	26.9±3.2	28.8±2.6	27.9±3.3

To account for variations in brain size, hippocampal volume is expressed relative to intracranial volume (ICV). The resulting ratios were multiplied by 1000. Data are presented as mean ± S.D. (n = 6 per group). Abbreviations: CON, control; CUMS, chronic unpredictable mild stress; ESC, escitalopram.

Repeated-measures analysis of variance showed a significant effect of laterality for NAA/Cr [F(1, 22) = 15.633, *P* = 0.001] and Cho/Cr [F(1, 22) = 10.355, *P* = 0.004], and a laterality×stress interaction for Cho/Cr [F(2, 21) = 10.953, *P* = 0.003], as well as a laterality×stress×drug interaction for NAA/Cr [F(3, 20) = 22.020, *P* = 0.000] and MI/Cr [F(3, 20) = 6.115, *P* = 0.022]. Therefore, separate ANOVA's in each hemisphere for each metabolite ratio were undertaken (Table 2). There was a significant effect of stress on bilateral NAA/Cr [F(1, 22) = 4.056, *P* = 0.048 for the right; F(1, 22) = 5.617, *P* = 0.028 for the left] and a stress×drug interaction for right NAA/Cr [F(2, 21) = 13.370, *P* = 0.002]. In the right hippocampus, NAA/Cr was significantly lower in the CUMS+saline group (1.11±0.05) compared with the control+saline group (1.35±0.09) (*P* = 0.004). NAA/Cr was significantly increased by escitalopram treatment, with elevated NAA/Cr in CUMS+escitalopram group (1.29±0.10) compared with the CUMS+saline group (*P* = 0.047). In the left hippocampus, there was a decrease of NAA/Cr in CUMS+saline group (1.13±0.05) compared with the control+saline group (1.18±0.09), although this difference was not significant (*P* = 0.198). As for Cho/Cr, Glu/Cr and MI/Cr, neither stress nor drug had a significant effect on them.

**Table 2:** Effects of CUMS and escitalopram treatment on hippocampal ^1^H-MRS measurements.

Laterality	Metabolites	CON+saline	CON+ESC	CUMS+saline	CUMS+ESC
Left	NAA/Cr	1.18±0.09	1.22±0.08	1.14±0.06	1.13±0.03
	Cho/Cr	0.16±0.02	0.18±0.02	0.19±0.02	0.18±0.03
	Glu/Cr	1.17±0.14	1.18±0.26	1.09±0.18	0.94±0.16
	MI/Cr	0.68±0.04	0.80±0.21	0.67±0.10	0.67±0.04
Right	NAA/Cr	1.35±0.09	1.22±0.15	1.11±0.05**	1.29±0.10#
	Cho/Cr	0.20±0.02	0.21±0.04	0.19±0.02	0.18±0.01
	Glu/Cr	1.29±0.09	1.16±0.22	1.14±0.12	1.09±0.10
	MI/Cr	0.68±0.08	0.65±0.11	0.62±0.05	0.69±0.09

Data are presented as mean ± S.D. (n = 6 per group).

**P<0.005 vs. control+saline group,

# *P*<0.05 vs. CUMS+saline group. Abbreviations: CON, control; CUMS, chronic unpredictable mild stress; ESC, escitalopram; NAA, N-acetylaspartate; Cr, creatine; Cho, choline-containing compounds; Glu, glutamate; MI, Myo-inositol.

### The relationship between behavioural tests and hippocampal neurochemistry

**Figure 3:**
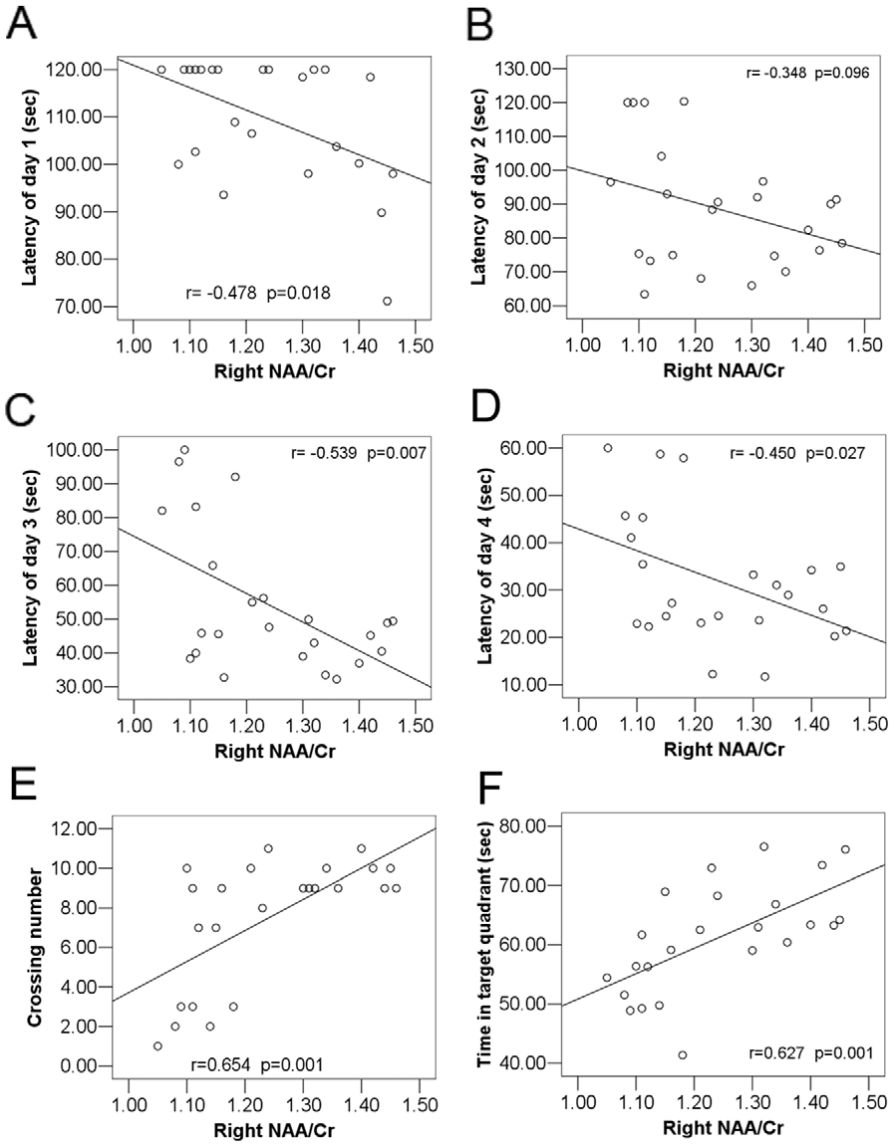
Correlation analysis between NAA/Cr of the right hippocampus and the learning and memory test. (A–D) In the acquisition trials, a significant negative correlation was found between NAA/Cr of right hippocampus and the latency to find platform on day 1 (r = −0.478, *P* = 0.018), day 3 (r = −0.539, *P* = 0.007) and day 4 (r = −0.450, *P* = 0.027), although the correlation of day 2 (r = −0.348, *P* = 0.096) was not significant. (E) A significant positive correlation was shown between NAA/Cr of right hippocampus and the crossing number of platform (r = 0.654, *P* = 0.001). (F) There is a strong positive correlation between NAA/Cr of right hippocampus and the time spent in the target quadrant (r = 0.627, p = 0.001).

Considering that the hippocampal neurochemistry alteration induced by CUMS and chronic escitalopram treatment was primarily in NAA/Cr, correlations between behavioural data and bilateral NAA/Cr were performed. Across all subjects, there was a significant negative correlation between NAA/Cr of the right hippocampus and the latency to find the platform in the acquisition trials of the Morris water maze test on day 1 (r = −0.478, *P* = 0.018), day 3 (r = −0.539, *P* = 0.007) and day 4 (r = −0.450, *P* = 0.027). The correlation of data from day 2 (r = −0.348, *P* = 0.096) was not significant (Fig. 3A–D). In addition, there was a significant positive correlation between NAA/Cr of the right hippocampus and the crossing number of platform (r = 0.654, *P* = 0.001) and the time in target quadrant (r = 0.627, *P* = 0.001) in the probe trial of the Morris water maze test (Fig. 3E–F). However, when the 4 groups were examined separately, the above correlation did not remain significant. In the left hippocampus, the correlations between NAA/Cr and the behavioural tests showed a similar trend, although the correlations did not reach statistical significance. We did not find significant correlations between right NAA/Cr and most indices of depression [sucrose preference at week 2 (r = 0.187, *P* = 0.382), immobility time at week 2 (r = −0.199, *P* = 0.352) and week 6 (r = −0.327, *P* = 0.119)], except for sucrose preference at week 6 (r = 0.471, *P* = 0.020). As for the left hippocampus, we found no significant correlations between NAA/Cr and the indices of depression mentioned above.

## Discussion

In the present study, we demonstrated that CUMS had a dramatic influence on spatial cognitive performance in the Morris water maze task, while the repeated administration of escitalopram significantly ameliorated the cognitive deficits. Furthermore, CUMS rats exhibited marked reduction of the hippocampus NAA, whereas administration of escitalopram to stressed rats prevented such metabolite reductions. However, neither stress nor escitalopram influenced hippocampus volume. Of note, the learning and memory alterations of the rats were associated with right hippocampal NAA.

Many studies have reported memory deficits in depressed subjects. An effect size analysis of cognitive functioning in 726 patients with major depressive disorder (MDD), conducted using meta-analytic principles, found that the type of memory task most affected by depression was recollection [25]. Recollection memory is analogous to explicit or declarative memory, requiring the conscious recall of specific facts or events. The present experiments showed a deficit of spatial memory in rats exposed to chronic stress, supporting the hypothesis that depressed subjects show differential impairment on memory tasks that are dependent on the hippocampus. Our results are consistent with a previous report showing a deficit of spatial memory in the T-maze following chronic stress in mice [26] and the findings of spatial memory deficits in other animal models of depression [5]–[6]. Furthermore, we found a reversal of memory deficits in animals administered the antidepressant escitalopram. This is consistent with the effects of psychotherapeutic treatments directed at changing cognitive processes [27] and is further supported by studies showing that antidepressant therapy improves cognitive function in both humans and animals [28]–[32].

The peak at 2.02 ppm, which emerges as the main peak of the ^1^H-MRS spectra from the normal brain, is prominently attributable to NAA, which is synthesised only in neurons of the adult central nervous system and is commonly considered to be a putative neuronal marker. There have been some reports indicating a decrease of NAA resonance in the hippocampus, frontal lobe, and basal ganglia of depressed subjects [33]. The present study found significantly decreased NAA in the hippocampus of chronically stressed animals, suggesting the loss of neuronal integrity in the hippocampus induced by chronic stress. This finding is similar to that of Czeh et al. who used a chronic psychosocial stress paradigm in male tree shrews and found that 1 month of stress could reduce the concentrations of NAA [34]. The possible mechanisms of decreased NAA in the hippocampus induced by chronic stress may involve an elevated glucocorticoid level. It has been shown that chronic physical or psychological stressors can provoke the secretion of glucocorticoid [35]. Numerous observations have demonstrated that glucocorticoid treatment inhibits neurogenesis in the dentate gyrus (DG) [10], induces arrest of the neural cell cycle [36] and apoptosis in neuronal progenitors [37] and initiates dendritic atrophy, synaptic loss, even cell apoptosis, among hippocampal neurons [38]–[40], finally resulting in neuronal dysfunction and loss as indicated by decreased NAA in hippocampus.

Recently, increasing evidence has indicated that chronic treatment with SSRIs may reverse the inhibition of neurogenesis [41] and the impairment of synaptic plasticity and dendritic arborisation in the hippocampus induced by glucocorticoids [42]. In addition, SSRIs may reverse the delay of the differentiation of neuronal progenitors into mature neurons [43] as well as protect against the death of neuronal cells [44] and re-establish granule cell density of the hippocampus. These findings are consistent with the effect of a reversal of NAA decrease upon treatment with escitalopram observed in the present study.

There have been studies showing a relationship between NAA and memory level in both normal subjects and patients with neuropsychiatric disorders [45]–[50]. In keeping with this, our study demonstrated that the learning and memory alterations of the animals were associated with hippocampal NAA. This argues that NAA is a sensitive measure of hippocampal neurons that are crucial for performance on tasks of memory. In addition, Block et al. found that clinical response to pharmacological treatment is associated with an increase in hippocampal NAA in patients with unipolar major depressive episodes [51]. Consistent with this, our study demonstrated that, as one component of the response to treatment, the improvement in learning and memory of the animals was associated with an increase in hippocampal NAA.

In most neuroimaging studies, regions of interests (ROIs) have usually beenmanually traced. This method is associated with many disadvantages including the relatively low objectivity and replicability as well as high labour and time intensity. Therefore, we collaboratively developed a new atlas-based method utilising brain template and digital atlas to trace the hippocampus automatically while simultaneously preserving the individual anatomical characteristics [52]. In the present study, we found no hippocampal volume differences as an effect of CUMS or escitalopram treatment. This is similar to our negative finding in another animal model of depression, maternal separation, which simulated early life stress [53]–[54]. While several clinical and preclinical studies have reported diminished volume of the hippocampus in depressed subjects, still others have reported larger or unchanged hippocampal volume in depression [11]. This apparent inconsistency may be due to differences in depressive burden, treatment history, and age at onset of depression as well as heterogeneity in the causes of depression. Other studies have reported reduced NAA in the hippocampus of posttraumatic stress disorder (PTSD) patients in the absence of hippocampal volume loss [45]. Along with the ^1^H-MRS findings, these results suggest that NAA may be a more sensitive measure of the integrity of the hippocampus than hippocampal volume.

Due to several limitations, the present study must be considered preliminary. Firstly, it relied on Cr as an internal signal standard, although Cr changes have been reported in depression in some studies [33]. Furthermore, even though the position of the VOI was carefully selected, we were restricted to a cubic voxel by MRI coil available in order to obtain high signal-to-noise ratios with limited scan time. Thus, the spatial resolution of ^1^H-MRS inevitably included some tissue outside the hippocampus, which may have introduced spurious metabolite variations. However, the lack of volume change in the hippocampus rules this out as a potential contributor to changes in the NAA ratio.

To the best of our knowledge, this is the first animal study examining the relationship between hippocampal neurochemistry and learning and memory in depression using non-invasive imaging technique MRS. These findings raise the possibility that low hippocampal NAA may be an underlying indicator of increased risk of learning and memory deficits in depression. Future studies will be needed to further examine learning and memory correlates of brain volume and chemistry, using more sensitive measures and moving beyond the hippocampus to examine additional regions of the brain that are involved in cognition.

## Materials and Methods

### Animals

Experiments were performed on adult male Sprague–Dawley rats (Animal House Center, Southeast University, China) weighing 200 g. Animals were housed 4 to a cage with food and water available *ad libitum*. All rats were kept on a 12 h light–dark cycle (lights on at 7 a.m.) in the same colony room, with constant temperature (21±2°C) and humidity (55±5%). All experiments were conducted in accordance with the National Institutes of Health Guide for the Care and Use of Laboratory Animals, and the experiments were approved by Jiang Su Animal Care and Use Committee.

### Experimental procedure and drug treatment

**Figure 4:**
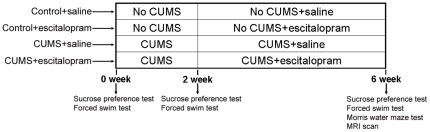
Schematic representation of animal group assignments and behavioural tests. Rats were randomly divided into 4 groups: control+saline, control+escitalopram, CUMS+saline and CUMS+escitalopram (n = 6 per group). Two weeks after the beginning of the CUMS regimen, rats received escitalopram (10 mg/kg, i.p.) or saline treatment once a day for another 4 weeks while the CUMS procedure was continued. Sucrose preference test and forced swimming test were measured before stress (0 week), before drug administration (2 week) and at the end of the experiment (6 week). Morris water maze and MRI scan were carried out at the end of the experiment.

Fig. 4 depicts how the experiment was conducted. The rats were randomly divided into 4 groups: control+saline, control+escitalopram, CUMS+saline and CUMS+escitalopram (n = 6 per group). Two weeks after the beginning of the CUMS regimen, rats received escitalopram (10 mg/kg, i.p.) or saline treatment once a day for 4 weeks. A sucrose preference test and a forced swimming test were administered at 3 time points: before stress (0 week), before drug administration (2 week) and at the end of the experiment (6 week). The Morris water maze test and MRI scans were carried out at the end of the experiment. The same cohort of animals was used throughout the study.

### CUMS procedure

The CUMS procedure contained 9 different stressors randomly arranged day and night across 42 consecutive days: 20 h food and water deprivation, 18 h water deprivation, 17 h of 45° cage tilt, overnight illumination, 21 h wet cage, 5 min swimming in water at 4°C, 30 min on a 160 Hz rocking bed, 1 min tail pinch and 2 h immobilisation. The behavioural tests were performed and scored by trained and experienced observers who were blind to the condition of the animals.

### Sucrose preference test

In the sucrose preference test, the animals were allowed to consume water and a 1% sucrose solution for 1 h after 20 h food and water deprivation, following 48 hours of exposure to both water and sucrose solution. The positions of the two bottles (right/left) were varied randomly across animals and were reversed after 30 min. The sucrose preference was calculated according to the following ratio: sucrose preference (%) = [sucrose intake (g)/sucrose intake (g)+water intake (g)]×100% [55]. The results reflect mean values of daily tests over three days.

### Forced swim test

For the forced swim test, a method used in previous research [56] was followed. Briefly, rats were forced to swim individually in a cylindrical glass container (40 cm height, 30 cm diameter) that contained tap water (25±1°C) of 28 cm depth. Then, 24 h after pre-swimming for 15 min, rats were subjected to a 5 min swimming test. All test sessions were recorded with a video camera from the opposite side of the cylinder. Periods of time when a rat was passively floating in the water, exerting minimal activity except respiration, was measured as immobility.

### Morris water maze test

The water maze was a black circular pool (160 cm in diameter) filled with opaque water (30 cm depth) at 25±1°C. An escape platform (11 cm in diameter) was placed in the middle of one of the quadrants (1 cm below the water surface). The behaviour of the animal was monitored with a video camera mounted in the ceiling above the centre of the pool. In the acquisition trials, the rats were trained for 120 s per trial and 4 trials per day starting at four different positions with 30 min intervals for 4 consecutive days. Each trial began with the rat in the pool facing the sidewalls. If the rat failed to escape within 120 s, it was guided to the platform by the experimenter. When the rat escaped onto the platform, the rat was allowed to stay on the platform for 30 s before being returned to its home cage. The hidden platform was removed on day 5, and memory retrieval was examined by a probe trial that lasted for 180 s. The escape latency in the acquisition trials, the times of crossing the platform position and the time spent in the target quadrant in the probe test were recorded by a computerised video tracking system.

### MRI and proton MRS (^1^H-MRS) acquisition

MRI was undertaken on a 7.0 T animal MRI scanner (70/16 PharmaScan, Bruker Biospin GmbH, Germany) using a 38 mm birdcage rat brain quadrature resonator for radiofrequency transmission and reception. Rats were anesthetised using inhaled isoflurane/O_2_ (3% for induction and 1.5–2% for maintenance). During the MRI scan, the rat was prostrated on a custom-made holder to minimise head motion while respiration was maintained at a rate of 50 breaths/min.

Scout T_2_-weighted imaging (T_2_WI) in three planes with a fast spin echo (FSE) pulse sequence was first acquired to control rat head positioning. Next, a coronal T_2_WI scan was acquired using rapid-acquisition relaxation-enhancement (RARE) pulse sequence with the following parameters: field of view = 4.5 cm×4.5 cm, matrix size = 256×256, repetition time (TR) = 3500 ms, echo time (TE) = 12 ms, slice thickness = 1.0 mm, slice gap = 1.5 mm, and acquisition time = 1 min 24 s.

**Figure 5:**
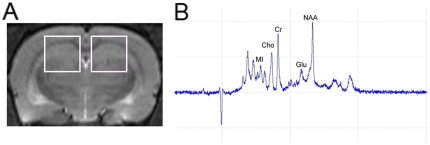
Illustration of the voxel to the site of hippocampus for ^1^H-MRS was presented, with associated magnetic resonance spectrographic imaging spectrum. (A) Left and right hippocampi are indicated by the white rectangles in coronal T_2_-weighted imaging of rat brain. (B) Typical hippocampal ^1^H-MRS with marked Myo-inositol (MI), choline-containing compounds (Cho), creatine and phosphocreatine (Cr), glutamate (Glu) and *N*-acetylaspartate (NAA) peaks.

For single-voxel ^1^H-MRS, the volume of interest (VOI, 3.5 mm×3.5 mm×3.5 mm) was placed over the hippocampal region in the coronal T_2_W images centred approximately at Bregma −3.8 mm of the standard rat brain atlas (Fig. 5A). Point resolved spectroscopy (PRESS) sequence was used for signal acquisition, with TR = 2500 ms, TE = 20 ms, number of average = 512 and scan duration for each side = 21 min. In addition, the order of acquisition of the right and left hippocampal spectra alternated between animals scanned, to minimise the introduction of artifactual hemispheric differences. A typical spectrum is shown in Fig. 5B.

### Hippocampus volume measurements and ^1^H-MRS spectral processing

The coronal T_2_WI data were analysed using statistical parametric mapping (SPM5). The volume of hippocampus was extracted using our atlas-based region of interest (ROI) extraction method that was developed in-house; this extraction method is based on T_2_WI rat brain template and digital atlas [52]. To account for variations in brain size, hippocampal volume was normalized to intracranial volume (ICV), which was extracted based on the PCNN (Pulse Coupled Neural Network) method.

The *in vivo* ^1^H-MRS were processed using jMRUI software (version 3.0, http://www.mrui.uab.es/mrui) according to the methods described by Chan et al [57]–[58] and our previously reported paper [53]–[54]. In short, metabolite peak areas were determined using the quantum estimation (QUEST) method combined with a subtraction approach for background modelling after preconditioning. A simulated basis set was used to estimate peak areas. To reduce systematic variations between animals, we applied a relative quantification method using the creatine (Cr) peak as the internal spectral reference. NAA/Cr, Cho/Cr, Glu/Cr, and MI/Cr were statistically evaluated.

### Statistical analysis

Data were analysed using SPSS software, version 13.0 (SPSS Inc., Chicago), and values were presented as mean ± standard deviation (S.D.). The results were considered statistically significant if *P*<0.05. Two-way ANOVA was applied by the Bonferroni testing as post hoc analysis for further examination of group differences. Considering that hippocampal volume and metabolite ratios were measured in bilateral hippocampi and the acquisition trials of Morris water maze test were carried out on 4 consecutive days, repeated-measures analysis of variance was initially performed. Pearson correlations between the data from the Morris water maze test and hippocampal volume and metabolite ratios were performed.
